# Effectiveness of thin polyurethane film dressings in preventing acute radiation dermatitis during postmastectomy radiotherapy for breast cancer: A retrospective study

**DOI:** 10.1016/j.apjon.2025.100801

**Published:** 2025-10-11

**Authors:** Keiko Oishi, Daisuke Nakamura, Tatsuya Takeda, Miyuki Shinchi, Tomomi Ashikari

**Affiliations:** a NHO Nagasaki Medical Center, Nagasaki, Japan; b Nagasaki University Hospital, Nagasaki, Japan

**Keywords:** Acute radiation dermatitis, Polyurethane film, Postmastectomy radiotherapy, Breast cancer, Nursing care, Quality of life

## Abstract

**Objective:**

Acute radiation dermatitis (ARD) is a frequent and distressing toxicity in patients receiving postmastectomy radiotherapy (PMRT), often impairing comfort and treatment adherence. Simple, evidence-based preventive measures are therefore essential for improving skin tolerance and quality of life. This study aims to evaluate the effectiveness of thin polyurethane film (PUF) dressings in preventing ARD among patients with breast cancer undergoing PMRT.

**Methods:**

A retrospective analysis was conducted in 38 patients who received PMRT at our institution between April 2019 and June 2024. Patients were divided into two groups: those who received full-field PUF dressings from April 2021 onward and those who received standard skin care between April 2019 and March 2021. The severity of ARD was graded using the Common Terminology Criteria for Adverse Events v5.0 (CTCAE v5.0). Both univariate and multivariate logistic regression analyses were performed to identify independent predictors of severe dermatitis.

**Results:**

Patients in the PUF group exhibited a significantly lower incidence of severe ARD compared with those receiving standard care. Multivariate analysis identified PUF use as an independent protective factor against severe dermatitis (odds ratio ​= ​0.18, 95% confidence interval ​= ​0.04–0.72, *P* ​= ​0.006). Age, body mass index, and chemotherapy were not significantly associated with ARD severity.

**Conclusions:**

Thin PUF dressings significantly reduced the risk of severe ARD in patients undergoing PMRT. This simple, noninvasive intervention alleviates discomfort, supports treatment continuity, and demonstrates strong clinical relevance in Asian practice settings, where supporting evidence remains limited compared with European trials. The findings highlight the importance of integrating PUF dressings into routine nursing care to enhance skin protection and maintain patients’ quality of life.

## Introduction

There is an increasing trend in the incidence of breast cancer among women in Asian countries, especially in China, Korea, and Japan. This is believed to be due not only to the westernization of lifestyle, such as increased smoking and dietary changes toward high-fat, high-calorie diets, but also to metabolic and reproductive factors. Recent epidemiological studies have shown that high body mass index (BMI), hyperglycemia, and physical inactivity significantly contribute to the rising incidence of breast cancer. Furthermore, demographic and reproductive changes, including delayed childbearing, reduced parity, and shorter breastfeeding duration, are also recognized as important risk factors.^1^, ^2^, ^3^

Breast cancer is the most common cancer among women worldwide, and radiotherapy (RT) plays an essential role in its multidisciplinary management. Postmastectomy radiation therapy (PMRT) is considered the standard treatment for patients with advanced disease. Clinical trials and meta-analyses have demonstrated that PMRT reduces local and regional lymph node recurrence to approximately one-third to one-quarter and contributes to improved survival.^4^^,^^5^ According to the Japanese Breast Cancer Society Clinical Practice Guidelines, PMRT is particularly recommended for patients with large primary tumors (T3 or greater) or those with four or more positive axillary lymph nodes, as these groups derive the greatest benefit in terms of local control and overall survival.^6^ Epidemiological data from Japanese multi-institutional surveys indicate that the actual implementation rate of PMRT varies depending on stage and clinical practice. For example, among patients with T1–2 disease and positive nodes, the implementation rate was as low as approximately 20%.^7^ This indicates that adoption is relatively low in early-stage disease, and the actual implementation rate likely varies depending on disease stage and institutional practice, with higher use anticipated in patients meeting guideline-based indications such as T3 tumors or ≥ 4 positive nodes.

Acute radiation dermatitis (ARD) is the most frequent adverse event during breast cancer RT. Severe ARD is typically defined as Grade ≥ 2 or ≥ 3 by the Common Terminology Criteria for Adverse Events (CTCAE v5.0).^8^ According to the MASCC clinical practice guidelines, ARD occurs in up to 95% of patients receiving RT.^9^ Furthermore, a prospective cohort study by Xie et al. reported that Grade ≥ 3 ARD developed in 59.6% of patients, and Grade ≥ 4 in 17.8%.^10^ Such severe reactions cause pain, exudation, and may even lead to treatment interruptions, significantly impairing patients' quality of life (QOL).^11^ In addition, ARD often restricts patients’ ability to wear regular clothing, perform daily activities, and may negatively affect body image and social interactions, further diminishing their overall well-being.^12^

At our institution, thin polyurethane film (PUF) has been used for over a decade to protect small skin markings during RT. Interestingly, areas covered by these 3–5 cm films showed visibly less severe dermatitis compared with surrounding irradiated skin once the films were removed. This observation prompted the hypothesis that applying PUF over the entire radiation field might reduce the severity of ARD. Therefore, we conducted this study to evaluate the protective effect of PUF on ARD in breast cancer patients undergoing PMRT.

## Methods

### Study design and participants

We retrospectively reviewed the medical records of 38 consecutive breast cancer patients who underwent postmastectomy RT (PMRT) at our institution between April 1, 2019, and June 30, 2024.

Patients were categorized into two groups according to the skin care protocol used during treatment. The PUF group comprised 22 patients who received a thin PUF dressing (Perme-roll Lite, Nitto) covering the entire irradiation field from the first day of RT. The Standard Care group included 16 patients treated prior to film implementation, who received conventional cleansing and moisturizing care without dressings.

### Sample size rationale

The total sample of 38 represents all eligible consecutive patients treated during the study period who met the inclusion criteria. As a retrospective exploratory study, the sample size was not determined by power analysis but was limited by the available patient population. Although relatively small, effect size estimates and multivariable adjustments were included to improve interpretability and analytical robustness.

### Inclusion and exclusion criteria

Eligible patients met the following criteria: (1) histologically confirmed breast cancer treated with total mastectomy; (2) planned RT dose ≥ 50 Gy; and (3) use of a 5-mm bolus up to 20 Gy. Patients were excluded if they discontinued PUF prematurely (e.g., due to skin irritation) or deviated from the prescribed treatment protocol.

### Radiotherapy procedures

RT was delivered using Elekta Synergy and Varian TrueBeam linear accelerators. Treatment planning was performed with Philips Pinnacle,^3^ Varian Eclipse, or Elekta Monaco systems, and treatment records were managed using the Elekta MOSAIQ record-and-verify system. Throughout the study period, the same radiation oncologists oversaw treatment planning. All plans adhered to the Japanese Breast Cancer Society Clinical Practice Guidelines, and no major protocol or technical modifications occurred that could introduce systematic bias. Nurses applied the PUF dressing on Day 1 and monitored adherence, exudation, and detachment daily. Dressings were replaced as needed and maintained until two weeks after completion of RT.

### Assessment and reliability assurance

In both groups, nurses assessed skin reactions and recorded ARD grades using the Common Terminology Criteria for Adverse Events (CTCAE v5.0). Patient-reported pain, itching, discomfort, and limitations in activities of daily living were documented.

Photographs of irradiated skin were systematically archived throughout the treatment course. Two radiation oncology nurses independently reviewed ARD grades, and any discrepancies were resolved by consensus to ensure assessment reliability.

### Statistical analysis

Data were extracted from electronic medical records and image archives. Statistical analyses were performed using IBM SPSS Statistics version 31.0.1.0. Categorical variables were analyzed using the chi-square or Fisher's exact test, and ordinal data (ARD grades) were compared with the Mann–Whitney *U* test. Logistic regression analysis was performed to identify independent predictors of Grade ≥ 2 ARD, with covariates including total radiation dose, age, electron use, 4-MeV beam use, and chemotherapy. Effect sizes (Cliff's delta and *r*) were calculated. Statistical significance was defined as *P* ​< ​0.05.

## Results

**Fig. 1:**
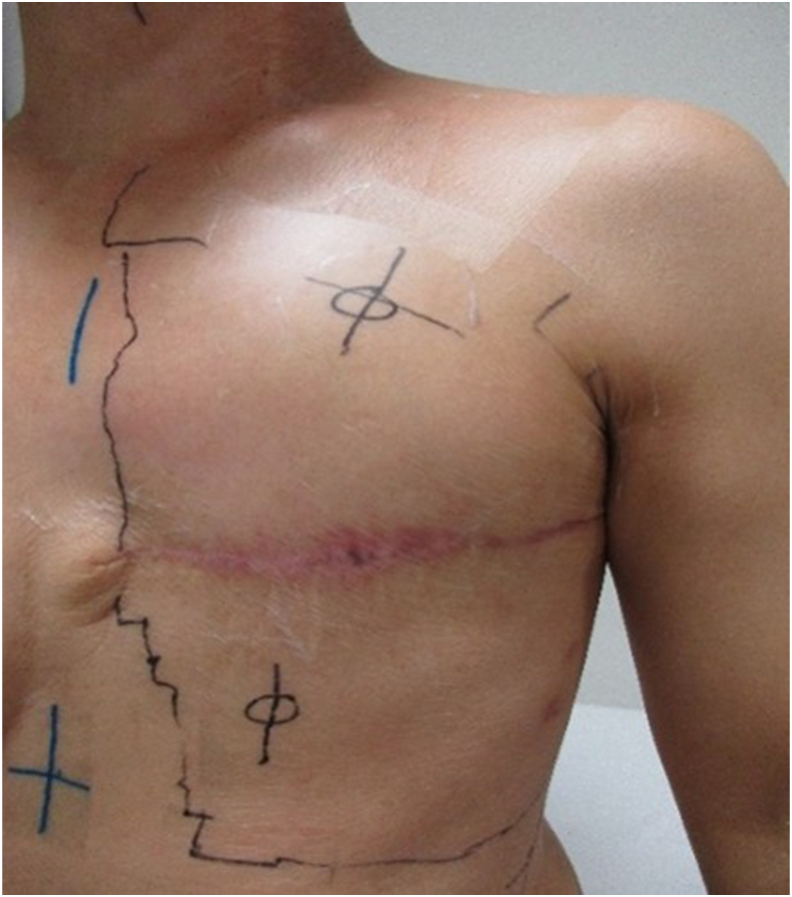
**Application of polyurethane film dressing at the initiation of radiotherapy in the polyurethane film group**. At the start of treatment, a thin, transparent polyurethane film is applied to cover the entire radiation field, as shown.

In this retrospective analysis, patients who underwent PMRT at our institution between April 1, 2019, and June 30, 2024, were divided into two groups according to the time period of treatment. The standard care group consisted of consecutive patients treated between April 2019 and March 2021, prior to the introduction of PUF in our department. The PUF group initially included 23 consecutive patients who received thin PUF dressing applied to the entire radiation field from the initiation of RT between April 2021 and June 2024. However, one patient was excluded due to non-compliance with the exclusion criteria, specifically premature discontinuation of PUF application caused by tape-related skin irritation. This left 22 patients for the final analysis. The difference in group sizes was not due to selection but reflected the number of consecutive patients treated during each period, ensuring comparability between groups. The application of the PUF dressings at the initiation of treatment is shown in Fig. 1.

**Fig. 2:**
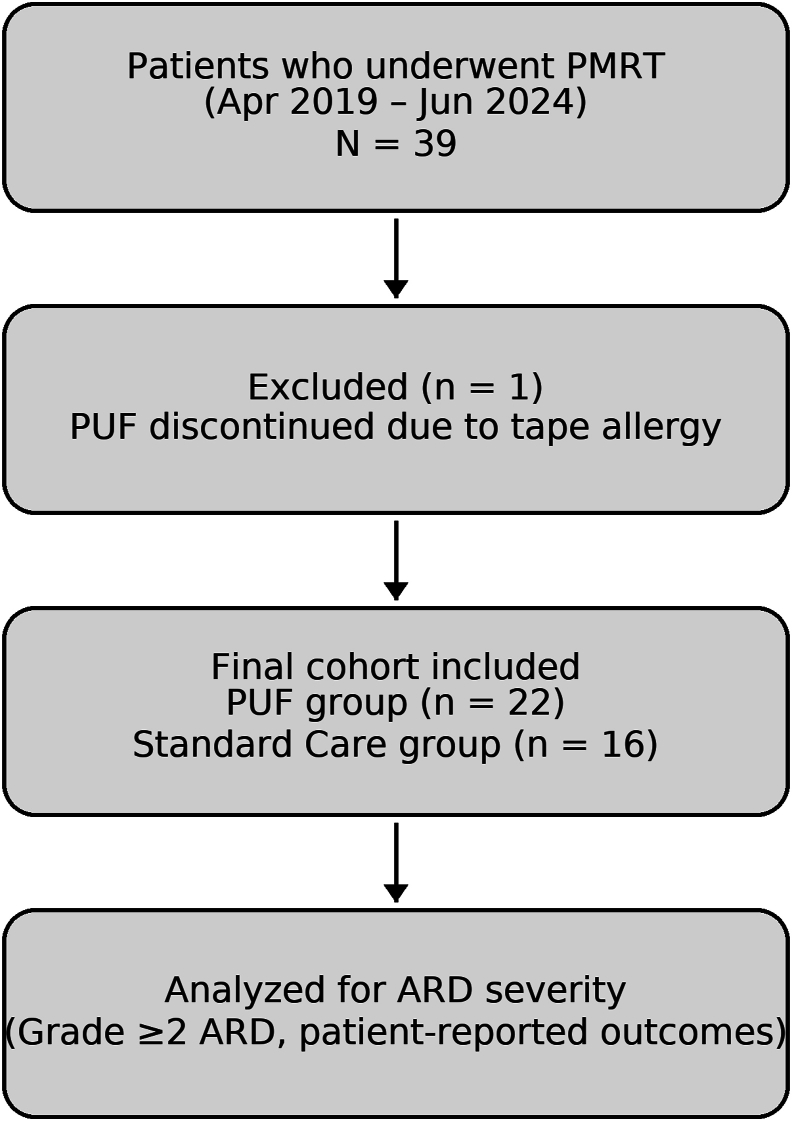
**STROBE flow diagram of patient selection**. Of 39 patients screened, one was excluded due to premature discontinuation of PUF caused by tape allergy. A total of 38 patients were included for analysis: 22 in the PUF group and 16 in the Standard Care group. PUF, polyurethane film; ARD, acute radiation dermatitis.

The study selection process is summarized in the STROBE flow diagram (Fig. 2). Of the 39 patients initially screened during the study period, one was excluded due to tape-related irritation resulting in premature removal of PUF. Accordingly, 38 patients met the inclusion criteria and were analyzed: 22 in the PUF group and 16 in the Standard Care group.

The Standard Care group comprised 16 patients who received standard skin care, including cleansing and moisturizing without any PUF application, from April 2019 to March 2021. All of the subjects received a 5 mm tissue-equivalent bolus from the start of irradiation up to 20 Gy. The Standard Care group consisted of patients ranging in age from 42 to 81 years, with a median age of 53 years, and a median irradiation dose of 50 Gy. The PUF group included patients aged 30–86 years, with a median age of 56 years, and a median irradiation dose of 60 Gy. The patient characteristics are presented in Table 1.

**Table 1:** Patient characteristics in the polyurethane film and Standard care groups.

Characteristics	PUF group (*n* ​= ​22)	Standard care group (*n* ​= ​16)
Age (median, range)	56 (30–86)	53 (42–81)
Radiation dose (Gy, median)	60	50
Bolus application (20 Gy, 5 mm)	Yes	Yes

The table summarizes the age distribution, radiation dose, and bolus application of each group. The median values and ranges are shown where applicable. PUF, polyurethane film.

Regarding the severity of ARD, in the PUF group, 72.7% of the patients in the PUF group experienced Grade 1 (mild erythema and itching), 22.7% had Grade 2 (localized exudate accumulation requiring ointment treatment), and 4.5% developed Grade 3 (extensive moist desquamation and contact bleeding). In contrast, the Standard Care group, 18.8%, 56.3%, and 25.0% experienced Grade 1, 2, and 3 ARDs, respectively. The results are presented in Table 2.

**Table 2:** Distribution of acute radiation dermatitis (ARD) severity in each group.

Grade	Polyurethane film group (*n* ​= ​22)	Standard care group (*n* ​= ​16)
Grade 1 mild erythema and itching	16 (72.7%)	3 (18.8%)
Grade 2 localized exudate accumulation requiring ointment treatment	5 (22.7%)	9 (56.3%)
Grade 3 extensive moist desquamation and contact bleeding	1 (4.5%)	4 (25.0%)

This table displays the number and percentage of patients with different CTCAE grades of ARD in both the polyurethane film and Standard Care groups. CTCAE, Common Terminology Criteria for Adverse Events.

Statistical analysis revealed a significant difference between the groups (*P* ​= ​0.004 by the chi-square test; *P* ​= ​0.002 by the Mann–Whitney *U* test), indicating that PUF application was associated with a lower incidence of severe ARD. The results are presented in Table 3.

**Table 3:** Summary of statistical analysis comparing the severity of acute radiation dermatitis (ARD) between the PUF group and the Standard care group.

Test	Statistic	*P*-value	Effect size
Chi-square test	–	0.004∗∗	–
Mann–Whitney *U* test	*U* ​= ​75.5, *Z* ​= ​−3.275	0.002∗∗	*r* ​= ​0.53 (large effect)
Fisher's exact test	–	< 0.01∗∗	–
Cliff's delta	*δ* ​= ​−0.48	–	Large effect size

The Chi-square test and Fisher's exact test revealed a significant difference in ARD grade distribution between the two groups. The Mann–Whitney *U* test demonstrated a statistically significant difference in ordinal severity scores, with a large effect size (*r* ​= ​0.53). Cliff's delta indicated a large non-parametric effect size (*δ* ​= ​−0.48), further supporting the efficacy of the polyurethane film dressing in reducing ARD severity. ∗*P* ​< ​0.05, ∗∗*P* ​< ​0.01. PUF, polyurethane film.

A multivariate binary logistic regression analysis was performed, including PUF application, 4 MeV photon use, electron beam use, and concurrent chemotherapy as covariates, to identify independent predictors of Grade ≥ 2 ARD. Logistic regression analyses were performed. First, univariate analyses were conducted for each potential factor, followed by a multivariate binary logistic regression model including PUF application, 4 MeV photon use, electron beam use, and concurrent chemotherapy to identify independent predictors of Grade ≥ 2 ARD. In the multivariate logistic regression analysis, the use of PUF was independently associated with a significantly reduced risk of developing Grade ≥ 2 ARD (*P* ​= ​0.006). Other variables, including radiation dose, age, electron beam use, 4 MeV photon energy, and concurrent chemotherapy, were not statistically significant predictors of ARD. Electron beam irradiation showed a non-significant trend toward an increased risk (*P* ​= ​0.065). Table 4 presents the results of the logistic regression analysis.

**Table 4:** Logistic regression analysis of factors associated with ≥ Grade 2 ARD.

Variable	Univariate OR (95% CI)	*P*-value	Multivariate OR (95% CI)	*P*-value
PUF group	0.087 (0.018–0.415)	**0.002∗∗**	0.022 (0.001–0.328)	**0.006∗∗**
Radiation dose (Gy)	0.936 (0.833–1.053)	0.272	0.709 (0.439–1.144)	0.159
Age	0.964 (0.914–1.017)	0.177	0.946 (0.872–1.027)	0.183
Electron beam	1.543 (0.422–5.639)	0.512	67.724 (0.769–5962.703)	0.065
4 MeV photon	3.923 (0.678–22.705)	0.127	5.971 (0.297–119.973)	0.243
Chemotherapy	2.462 (0.514–11.799)	0.260	10.117 (0.555–184.293)	0.118

Univariate analysis represents models with each factor entered individually. Multivariate analysis represents the model with all variables (PUF group, radiation dose, age, electron beam, 4 MeV photon, chemotherapy) entered simultaneously. PUF group was identified as an independent protective factor against ​≥ ​Grade 2 ARD. Analyses were performed using IBM SPSS Statistics, Version 31.0.1.0 (IBM Corp., Armonk, NY, USA). ∗*P* ​< ​0.05, ∗∗*P* ​< ​0.01. PUF, polyurethane film; OR, Odds Ratio; CI, Confidence Interval; ARD, acute radiation dermatitis.

**Fig. 3:**
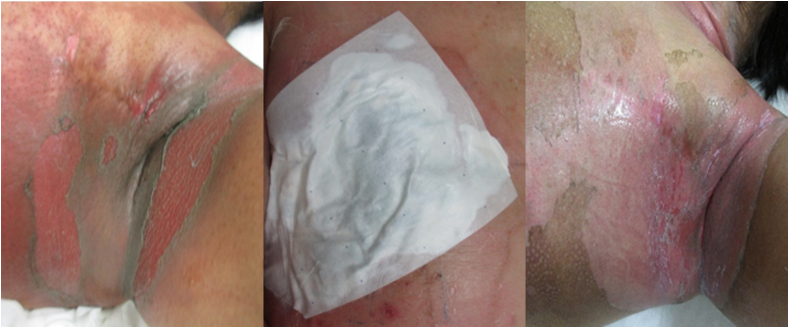
**Skin condition in the Standard Care group following 60 Gy irradiation**. Photographs illustrate severe ARD, including moist desquamation and exudation. Findings are shown at treatment completion, day 14, and day 21 after radiotherapy, consistent with CTCAE v5.0 grading of ARD. CTCAE, Common Terminology Criteria for Adverse Events; ARD, acute radiation dermatitis.

**Fig. 4:**
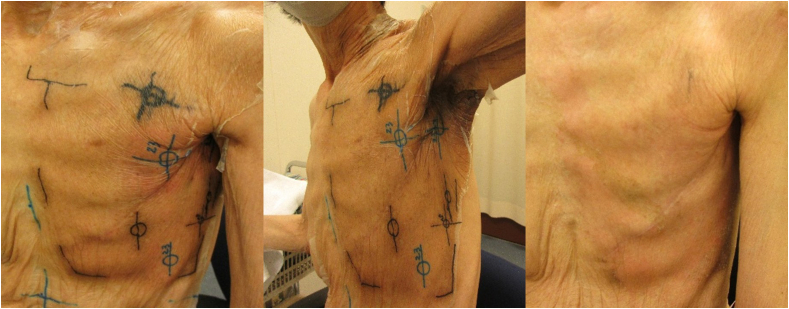
**Skin condition in the polyurethane film group following 60 Gy irradiation**. Photographs illustrate representative skin findings at treatment completion, day 14 (before film removal), and after removal. No moist desquamation or severe erythema was observed, indicating effective prevention of ARD consistent with CTCAE v5.0 criteria. CTCAE, Common Terminology Criteria for Adverse Events; ARD, acute radiation dermatitis.

Fig. 3, Fig. 4 illustrate the changes in skin condition from the day RT was completed in both the groups.

In the Standard Care group, moist desquamation was observed even before completion of RT, necessitating intervention with a topical ointment. After RT, patients continued to require daily clinic visits for cleansing and ointment application and experienced pain that interfered with their daily lives. Patients in this group frequently reported painful friction under the axilla, discomfort from exudate, and difficulty in performing daily activities.

Conversely, patients in the PUF group reported no pain and were able to maintain normal daily activities without the need for clinic visits, even after the completion of RT. They described minimal pain, little restriction in arm movement, reduced discomfort during bathing, and less interference with clothing. The PUF dressing was removed 14 days after treatment, revealing clear skin with no further intervention necessary. Notably, differences between the two groups were primarily observed in regard to pain levels, which significantly affected the QOL of the patients.

## Discussion

Previous studies have reported that 92.6% of patients undergoing PMRT develop erythema or desquamation, leading to impaired skin barrier function.^8^ Given that the present study targeted patients receiving high radiation doses with tissue-equivalent bolus use, severe ARD was expected.^13^

Comparison between standard care and PUF application showed a significant reduction in ARD severity in the PUF group. This suggests that the PUF helps prevent friction- and pressure-related damage by shielding the irradiated skin from external stimuli.

Previous studies have demonstrated that maintaining a moist environment promotes keratinocyte proliferation and migration, thereby accelerating re-epithelialization and wound healing.^14^^,^^15^ These reports support the hypothesis that moisture-retentive dressings can preserve skin barrier integrity and facilitate recovery after radiation-induced injury. In our study, the polyurethane film used (Perme-roll Lite)^16^ possesses transparency, vapor permeability, waterproof protection, flexibility, and adherence. These properties are consistent with those reported to reduce shear stress and mechanical irritation in prior studies.^17^ Such characteristics likely created a stable and protective skin microenvironment, which may explain why patients in the PUF group experienced reduced severity of ARD compared with those who received standard care.

Furthermore, evidence from randomized and observational studies has shown that polyurethane-based dressings, such as hydrofilms and Mepitel Film, reduce erythema and hyperpigmentation during breast RT.^12^^,^^17^ Our findings are consistent with these reports and suggest that similar benefits of PUF could also be observed in Japanese PMRT cohorts, where ARD tends to be more severe because of bolus use and high cumulative dose.

Importantly, ARD has a direct impact on patients’ daily lives. Patients with Grade 2 or higher ARD often experience burning sensations, itching, and pain, which not only impair skin integrity but also interfere with QOL. Prior reports indicate that ARD can cause bathing restrictions, movement-related pain, and sleep disturbances.^11^^,^^18^ In our cohort, patients in the standard care group frequently reported painful friction under the axilla, discomfort from exudate, and difficulty performing daily activities. Conversely, patients in the PUF group reported minimal pain, little restriction in arm movement, reduced discomfort during bathing, and less interference with clothing. These findings suggest that PUF application improved QOL by reducing pain-related limitations, maintaining comfort, and minimizing disruption of daily living.

Moreover, standard skin care regimens that rely on patient compliance with cleansing and moisturizing can be inconsistent.^19^^,^^20^ In contrast, PUF provides continuous protection independent of patient adherence, ensuring uniform preventive care throughout the course of treatment. This not only reduces the risk of severe ARD but also simplifies nursing management. Consistent protection may ease the emotional and physical burden on nurses, enhancing overall care quality.^20^^,^^21^

However, excessive moisture retention under PUF could pose risks such as maceration or infection susceptibility. In our study, Grade 2–3 cases in the PUF group were characterized by localized exudate, suggesting potential overhydration. Future research should evaluate optimal replacement intervals and duration of use to mitigate these risks.

### Limitations

The limitations of this study include its retrospective design and limited sample size, which restrict statistical precision. While multivariable logistic regression including radiation dose (50 Gy vs. 60 Gy), age, electron use, and chemotherapy confirmed that PUF was independently associated with reduced risk of Grade ≥ 2 ARD, residual confounding cannot be completely excluded. In particular, the approximately 10 Gy difference in prescribed dose between groups may reflect unmeasured differences in skin dose distribution or clinical decision-making. Although radiation dose was not a statistically significant independent factor in our model, subtle variations in treatment planning, supportive skin care, or other institutional practices may still have influenced outcomes. Therefore, these results should be interpreted with caution, and larger prospective studies with detailed dosimetric validation are warranted. Despite these limitations, this study suggests that the use of thin polyurethane film dressings is an effective and practical approach to reducing the incidence and severity of ARD in breast cancer patients undergoing PMRT. This approach not only improves skin integrity and patient QOL but also eases the workload on nurses and may contribute to improving the overall quality of care.

## Conclusions

Our study suggests that the application of PUF dressings may be effective in preventing severe ARD. This intervention facilitated improved treatment adherence, alleviated patient discomfort, reduced the burden on nursing staff, and contributed to the preservation of QOL in patients receiving PMRT. Importantly, multivariate logistic regression demonstrated that PUF was independently associated with a reduced risk of Grade ≥ 2 ARD, supporting its protective role even after adjusting for other potential confounders.

## CRediT authorship contribution statement

**Keiko Oishi:** Conceptualization, Methodology, Data Analysis, Writing – Original Draft. **Daisuke Nakamura:** Validation, Supervision, Writing – Review & Editing. **Tatsuya Takeda:** Data curation, Resources. **Miyuki Shinchi:** Investigation, Visualization. **Tomomi Ashikari:** Data collection, Project administration. All authors have read and approved the final version.

## Ethics statement

This retrospective study was approved by the Ethics Committee of the Nagasaki Medical Center (Approval No. 202430) and was conducted in accordance with the 1964 Helsinki Declaration and its later amendments or comparable ethical standards. Informed consent was waived by our Institutional Review Board because of the retrospective nature of our study. All patient data were anonymized prior to analysis.

## Data availability statement

The datasets generated and/or analyzed during the current study are not publicly available due to patient privacy and institutional policy but are available from the corresponding author upon reasonable request.

## Declaration of generative AI and AI-assisted technologies in the writing process

During the preparation of this work, the authors used ChatGPT (OpenAI, San Francisco, USA) for English language editing and proofreading. The authors reviewed and approved all content and take full responsibility for the publication.

## Funding

This study was supported by a research grant from the Yasuda Memorial Foundation and by an institutional research grant from Nagasaki Medical Center. The funders had no role in the study design, data collection, analysis, interpretation, or manuscript preparation.

## Declaration of competing interest

The authors declare no conflict of interest.
